# Supplementary figures and images for: Temporal shift in task factor influence across the stretch reflex
Source: PLoS One. 2026 Jun 1;21(6):e0350818. doi: 10.1371/journal.pone.0350818 (PMC13225632; doi:10.1371/journal.pone.0350818)

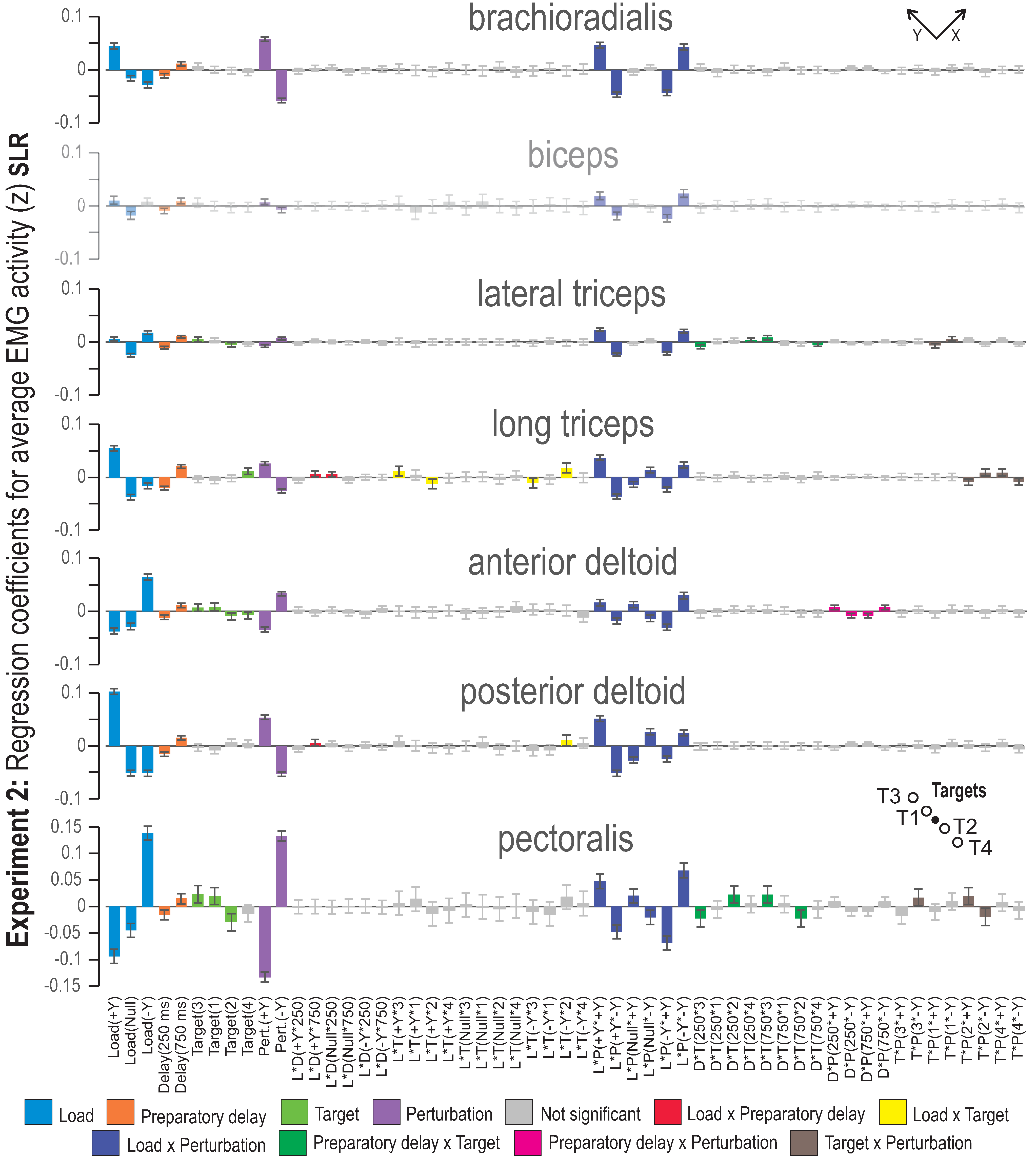

Supplement: S1 Fig — The -Y and +Y directions are the same as defined in Figure 1. Coloring represents factor type; main factors and two-factor interactions whose 95% confidence interval includes zero are indicated as not significant (i.e., grey bars). Biceps is faded to indicate that the MLR model is not significant. Abbreviations: L = load, D = preparatory delay, T = target, P = perturbation. (TIF) [file pone.0350818.s001.tif]

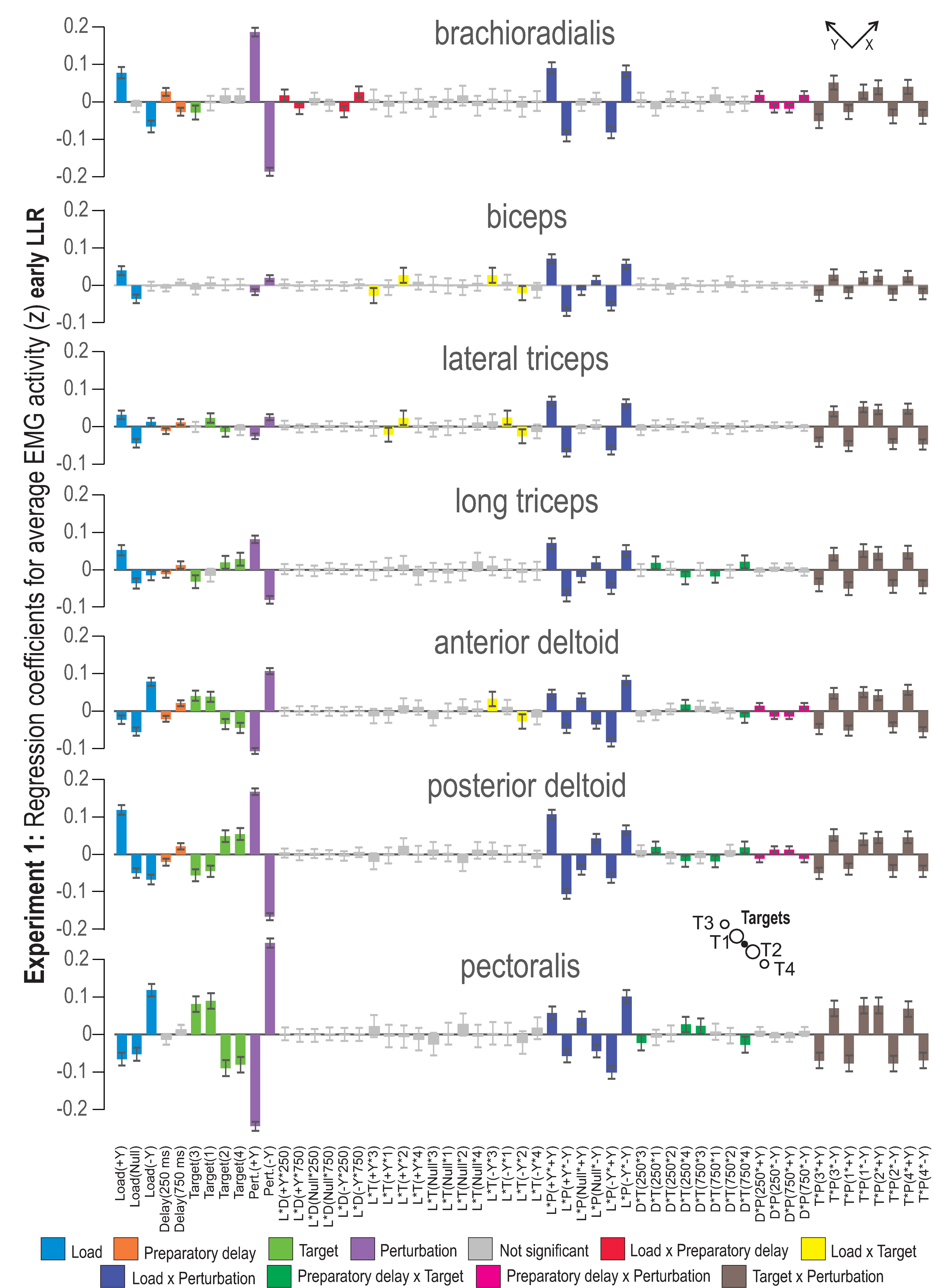

Supplement: S2 Fig — The -Y and +Y directions are the same as defined in Figure 1. Coloring represents factor type; main factors and two-factor interactions whose 95% confidence interval includes zero are indicated as not significant (i.e., grey bars). Abbreviations: L = load, D = preparatory delay, T = target, P = perturbation. (TIF) [file pone.0350818.s002.tif]

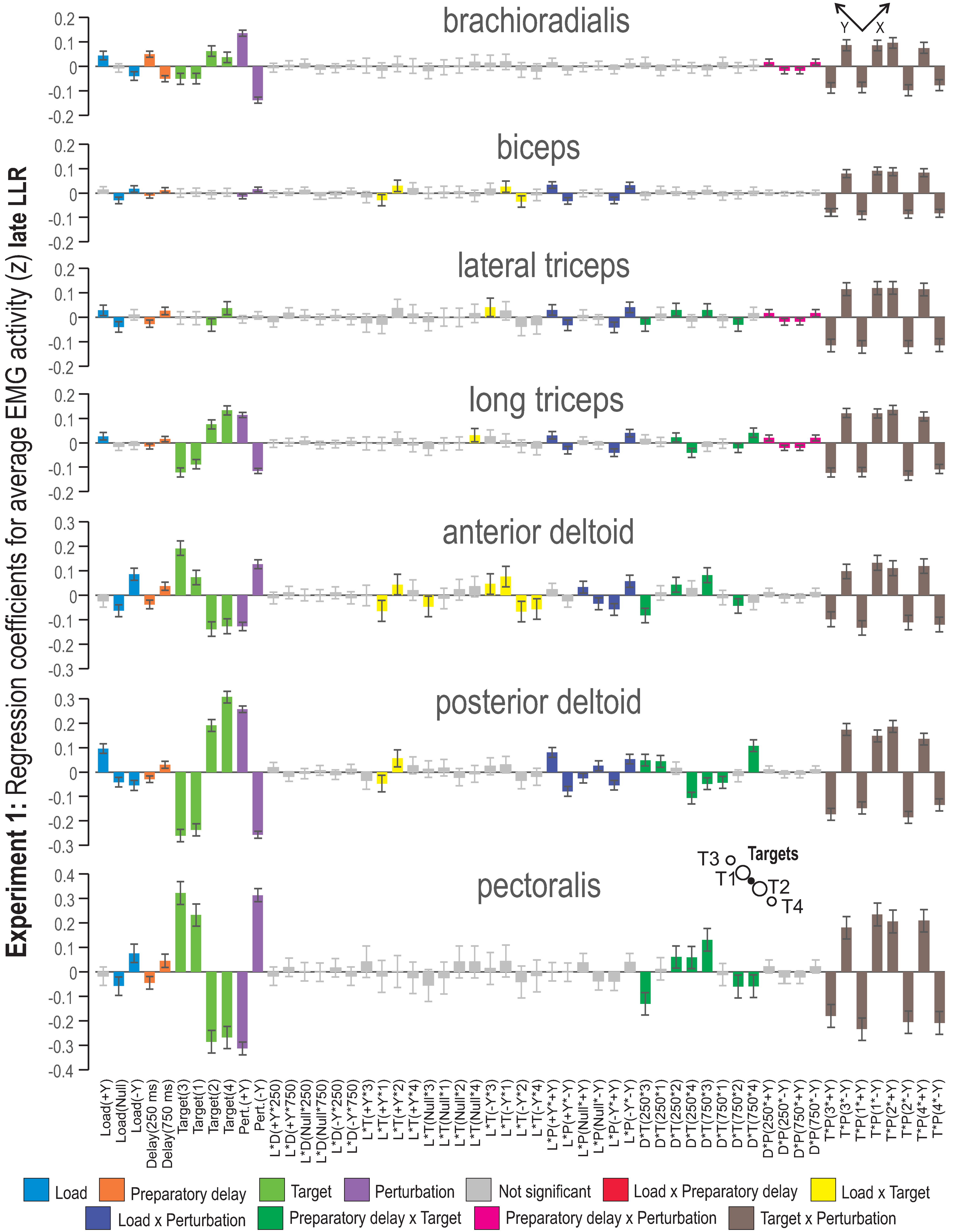

Supplement: S3 Fig — The -Y and +Y directions are the same as defined in Figure 1. Coloring represents factor type; main factors and two-factor interactions whose 95% confidence interval includes zero are indicated as not significant (i.e., grey bars). Abbreviations: L = load, D = preparatory delay, T = target, P = perturbation. (TIF) [file pone.0350818.s003.tif]
